# 3-Benzoyl-4-hydr­oxy-2*H*-1,2-benzothia­zine 1,1-dioxide

**DOI:** 10.1107/S1600536810009359

**Published:** 2010-03-17

**Authors:** Matloob Ahmad, Hamid Latif Siddiqui, Umar Farooq Rizvi, Saeed Ahmad, Masood Parvez

**Affiliations:** a Applied Chemistry Research Centre, PCSIR Laboratories Complex, Lahore 54600, Pakistan; b Institute of Chemistry, University of the Punjab, Lahore 54590, Pakistan; c Department of Chemistry, Gomal University, Dera Ismail Khan, NWFP, Pakistan; d Department of Chemistry, The University of Calgary, 2500 University Drive NW, Calgary, Alberta, Canada T2N 1N4

## Abstract

There are two mol­ecules in the asymmetric unit of the title compound, C_15_H_11_NO_4_S. The heterocyclic thia­zine rings in both mol­ecules adopt half-chair conformations with the S and N atoms displaced by 0.455 (4) and 0.254 (4) Å, respectively, in one mol­ecule, and 0.480 (4) and 0.224 (5) Å in the other, on opposite sides of the mean planes formed by the remaining ring atoms. The crystal structure is stabilized by inter­molecular N—H⋯O and C—H⋯O hydrogen bonds. In addition, intra­molecular O—H⋯O inter­actions are also present.

## Related literature

For the biological activity of 1,2-benzothia­zine derivatives, see: Ahmad *et al.* (2010 ▶); Lombardino *et al.* (1971 ▶, 1973 ▶). For the synthesis of benzothia­zine derivatives, see: Siddiqui *et al.* (2007 ▶). For comparison of bond distancess, see: Allen (2002 ▶). For related structures, see: Siddiqui *et al.* (2008 ▶)
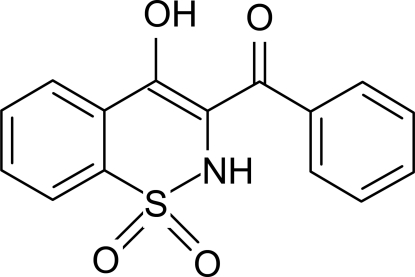


## Experimental

### 

#### Crystal data

C_15_H_11_NO_4_S
*M* *_r_* = 301.31
Monoclinic, 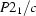

*a* = 13.8675 (4) Å
*b* = 7.6289 (2) Å
*c* = 25.7553 (9) Å
β = 102.4519 (12)°
*V* = 2660.66 (14) Å^3^
*Z* = 8
Mo *K*α radiation
μ = 0.26 mm^−1^
*T* = 173 K
0.12 × 0.11 × 0.08 mm

#### Data collection

Nonius KappaCCD diffractometer
Absorption correction: multi-scan (*SORTAV*; Blessing, 1997 ▶) *T*_min_ = 0.970, *T*_max_ = 0.980
10424 measured reflections
5971 independent reflections
5100 reflections with *I* > 2σ(*I*)
*R*_int_ = 0.034

#### Refinement

*R*[*F* ^2^ > 2σ(*F* ^2^)] = 0.048
*wR*(*F* ^2^) = 0.119
*S* = 1.09
5971 reflections
391 parameters
H atoms treated by a mixture of independent and constrained refinement
Δρ_max_ = 0.40 e Å^−3^
Δρ_min_ = −0.45 e Å^−3^

### 

Data collection: *COLLECT* (Hooft, 1998 ▶); cell refinement: *DENZO* (Otwinowski & Minor, 1997 ▶); data reduction: *SCALEPACK* (Otwinowski & Minor, 1997 ▶); program(s) used to solve structure: *SHELXS97* (Sheldrick, 2008 ▶); program(s) used to refine structure: *SHELXL97* (Sheldrick, 2008 ▶); molecular graphics: *ORTEP-3 for Windows* (Farrugia, 1997 ▶); software used to prepare material for publication: *SHELXL97*.

## Supplementary Material

Crystal structure: contains datablocks global, I. DOI: 10.1107/S1600536810009359/jh2134sup1.cif

Structure factors: contains datablocks I. DOI: 10.1107/S1600536810009359/jh2134Isup2.hkl

Additional supplementary materials:  crystallographic information; 3D view; checkCIF report

## Figures and Tables

**Table 1:** Hydrogen-bond geometry (Å, °)

*D*—H⋯*A*	*D*—H	H⋯*A*	*D*⋯*A*	*D*—H⋯*A*
N1—H1*N*⋯O8	0.84 (3)	2.30 (3)	3.093 (3)	159 (2)
O3—H3*O*⋯O4	0.97 (3)	1.55 (3)	2.466 (2)	155 (2)
O7—H7*O*⋯O8	0.96 (3)	1.62 (3)	2.510 (2)	153 (3)
C2—H2⋯O5^i^	0.95	2.57	3.310 (3)	135
C13—H13⋯O1^ii^	0.95	2.43	3.235 (3)	143
C14—H14⋯O8^ii^	0.95	2.48	3.396 (3)	162

Symmetry codes: (i) 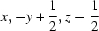; (ii) 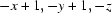.

**Fig. 1.**
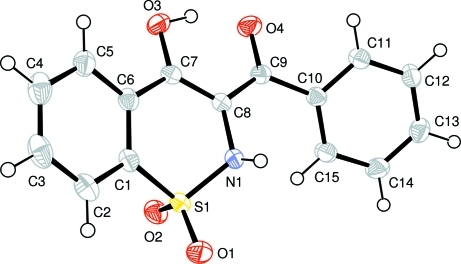
The molecule A of the title compound with the displacement ellipsoids plotted at 50% probability level (Farrugia, 1997).

**Fig. 2.**
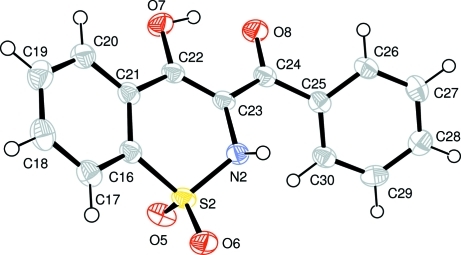
The molecule B of the title compound with the displacement ellipsoids plotted at 50% probability level (Farrugia, 1997).

**Fig. 3.**
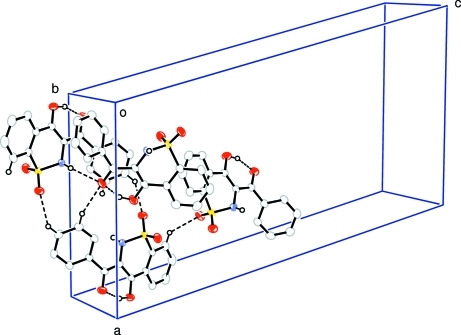
A part of the unit cell showing intermolecular and intrmolecular hydrogen bonds by dashed lines; the H-atoms not involved in H-bonds have been excluded for clarity.

## supplementary crystallographic information

### Comment

Benzothiazine derivatives, e.g., 4-hydroxy-2-methyl-2*H*-1,2-benzothiazine-3-carboxamides 1,1-dioxides, are potent anti-inflammatory agents (Lombardino *et al.*, 1971, 1973). In continuation of our research project on the development of new benzothiazine derivatives with bioactivity potential (Ahmad *et al.*, 2010; Siddiqui *et al.*, 2007), we report the synthesis and crystal structure of the title compound in this article.

The structure of the title compound is composed of two molecules, A (Fig. 1) and B (Fig. 2) in an asymmetric unit. The bond distances and angles are as expected (Allen, 2002) and agree with the cortresponding bond distances and angles reported in closely related compounds (Siddiqui *et al.*, 2008). The heterocyclic thiazine rings in both molecules adopt half chair conformation with atoms S and N displaced by 0.455 (4) and 0.254 (4) Å in molecule A and 0.480 (4) and 0.224 (5) Å, respectively, in molecule B, on the opposite sides from the mean planes formed by the remaining ring atoms.

The structure is stabilized by intermolecular hydrogen bonds of the types N—H···O and C—H···O. In addition, intramolecular interactions of the type O—H···O are also present consolidating the crystal packing; details have been provided in Tab. 1 and Fig. 3. It is intersing to note that N1 is involved in intermolecular and intramolecular interactions while N2 is devoid of any such interactions.

### Experimental

*N*-phenacylsaccharin (5.0 g, 16.6 mmoles) was added to a solution of sodium metal (2.7 g) in dry methanol (50 ml). The mixture was subjected to reflux for half an hour. The contents of the flask were cooled to room temperature and then poured on ice cold HCl (50 ml, 5%). Off white precipitates of the title compound were formed which were filtered off and were washed with excess distilled water. Crystals suitable for crystallographic study were grown from a solution of chloroform/methanol (4:1); yield = 3.5 g, 70%; m.p. = 429-430 K.

### Refinement

Though all the H atoms could be distinguished in the difference Fourier map the H-atoms bonded to C-atoms were included at geometrically idealized positions and refined in riding-model approximation with C—H = 0.95 Å; the H-atoms bonded to N and O were allowed to refine. The *U*_iso_(H) were allowed at 1.2*U*_eq_(parent atom). The final difference map was essentially featurless.

### Crystal data

**Table:**

C_15_H_11_NO_4_S	*F*(000) = 1248
*M**_r_* = 301.31	*D*_x_ = 1.504 Mg m^−^^3^
Monoclinic, *P*2_1_/*c*	Mo *K*α radiation, λ = 0.71073 Å
Hall symbol: -P 2ybc	Cell parameters from 5113 reflections
*a* = 13.8675 (4) Å	θ = 1.0–27.5°
*b* = 7.6289 (2) Å	µ = 0.26 mm^−^^1^
*c* = 25.7553 (9) Å	*T* = 173 K
β = 102.4519 (12)°	Block, yellow
*V* = 2660.66 (14) Å^3^	0.12 × 0.11 × 0.08 mm
*Z* = 8	

### Data collection

**Table:**

Nonius KappaCCD diffractometer	5971 independent reflections
Radiation source: fine-focus sealed tube	5100 reflections with *I* > 2σ(*I*)
graphite	*R*_int_ = 0.034
ω and φ scans	θ_max_ = 27.5°, θ_min_ = 2.0°
Absorption correction: multi-scan (*SORTAV*; Blessing, 1997)	*h* = −17→18
*T*_min_ = 0.970, *T*_max_ = 0.980	*k* = −9→9
10424 measured reflections	*l* = −33→33

### Refinement

**Table:**

Refinement on *F*^2^	Primary atom site location: structure-invariant direct methods
Least-squares matrix: full	Secondary atom site location: difference Fourier map
*R*[*F*^2^ > 2σ(*F*^2^)] = 0.048	Hydrogen site location: difference Fourier map
*wR*(*F*^2^) = 0.119	H atoms treated by a mixture of independent and constrained refinement
*S* = 1.09	*w* = 1/[σ^2^(*F*_o_^2^) + (0.0348*P*)^2^ + 2.7188*P*] where *P* = (*F*_o_^2^ + 2*F*_c_^2^)/3
5971 reflections	(Δ/σ)_max_ < 0.001
391 parameters	Δρ_max_ = 0.40 e Å^−^^3^
0 restraints	Δρ_min_ = −0.44 e Å^−^^3^

### Special details

**Table:**

Geometry. All esds (except the esd in the dihedral angle between two l.s. planes) are estimated using the full covariance matrix. The cell esds are taken into account individually in the estimation of esds in distances, angles and torsion angles; correlations between esds in cell parameters are only used when they are defined by crystal symmetry. An approximate (isotropic) treatment of cell esds is used for estimating esds involving l.s. planes.
Refinement. Refinement of *F*^2^ against ALL reflections. The weighted *R*-factor wR and goodness of fit *S* are based on *F*^2^, conventional *R*-factors *R* are based on *F*, with *F* set to zero for negative *F*^2^. The threshold expression of *F*^2^ > σ(*F*^2^) is used only for calculating *R*-factors(gt) etc. and is not relevant to the choice of reflections for refinement. *R*-factors based on *F*^2^ are statistically about twice as large as those based on *F*, and *R*- factors based on ALL data will be even larger.

### Fractional atomic coordinates and isotropic or equivalent isotropic displacement parameters (Å^2^)

**Table:**

	*x*	*y*	*z*	*U*_iso_*/*U*_eq_	
S1	0.26603 (4)	0.49396 (7)	−0.15139 (2)	0.02569 (13)	
S2	0.26612 (4)	0.00960 (7)	0.14780 (2)	0.02805 (13)	
O1	0.34849 (11)	0.3951 (2)	−0.16022 (6)	0.0392 (4)	
O2	0.26099 (11)	0.6780 (2)	−0.16221 (6)	0.0338 (4)	
O3	−0.00584 (11)	0.5375 (2)	−0.10052 (6)	0.0344 (4)	
H3O	0.0138 (19)	0.580 (3)	−0.0643 (12)	0.041*	
O4	0.08737 (11)	0.6136 (2)	−0.01058 (6)	0.0365 (4)	
O5	0.24183 (12)	0.1891 (2)	0.15448 (6)	0.0367 (4)	
O6	0.20854 (12)	−0.1254 (2)	0.16478 (6)	0.0362 (4)	
O7	0.49817 (12)	0.1805 (2)	0.07325 (7)	0.0386 (4)	
H7O	0.467 (2)	0.200 (4)	0.0367 (12)	0.046*	
O8	0.37534 (12)	0.1864 (2)	−0.01398 (6)	0.0392 (4)	
N1	0.25817 (13)	0.4634 (2)	−0.08997 (7)	0.0255 (4)	
H1N	0.2839 (18)	0.370 (3)	−0.0763 (10)	0.031*	
N2	0.26406 (13)	−0.0224 (2)	0.08502 (7)	0.0277 (4)	
H2N	0.2516 (18)	−0.123 (4)	0.0752 (10)	0.033*	
C1	0.15563 (15)	0.4015 (3)	−0.18693 (8)	0.0257 (4)	
C2	0.15101 (18)	0.3292 (3)	−0.23688 (9)	0.0333 (5)	
H2	0.2086	0.3204	−0.2511	0.040*	
C3	0.0605 (2)	0.2700 (3)	−0.26569 (9)	0.0401 (6)	
H3	0.0557	0.2224	−0.3002	0.048*	
C4	−0.02274 (19)	0.2799 (3)	−0.24437 (9)	0.0391 (6)	
H4	−0.0841	0.2378	−0.2643	0.047*	
C5	−0.01775 (17)	0.3508 (3)	−0.19421 (9)	0.0322 (5)	
H5	−0.0753	0.3561	−0.1799	0.039*	
C6	0.07235 (15)	0.4143 (3)	−0.16478 (8)	0.0254 (4)	
C7	0.07853 (15)	0.4912 (3)	−0.11193 (8)	0.0252 (4)	
C8	0.16798 (14)	0.5127 (3)	−0.07560 (8)	0.0237 (4)	
C9	0.16839 (15)	0.5735 (3)	−0.02290 (8)	0.0259 (4)	
C10	0.25834 (14)	0.5793 (3)	0.02069 (8)	0.0244 (4)	
C11	0.24653 (16)	0.5335 (3)	0.07145 (8)	0.0281 (4)	
H11	0.1836	0.4978	0.0765	0.034*	
C12	0.32640 (16)	0.5397 (3)	0.11451 (9)	0.0310 (5)	
H12	0.3182	0.5073	0.1489	0.037*	
C13	0.41781 (16)	0.5932 (3)	0.10724 (9)	0.0334 (5)	
H13	0.4724	0.5978	0.1367	0.040*	
C14	0.43023 (16)	0.6402 (3)	0.05692 (9)	0.0349 (5)	
H14	0.4931	0.6778	0.0523	0.042*	
C15	0.35116 (16)	0.6326 (3)	0.01342 (9)	0.0306 (5)	
H15	0.3600	0.6633	−0.0210	0.037*	
C16	0.39179 (16)	−0.0183 (3)	0.17731 (8)	0.0273 (4)	
C17	0.42052 (18)	−0.0878 (3)	0.22798 (9)	0.0325 (5)	
H17	0.3726	−0.1277	0.2466	0.039*	
C18	0.52034 (18)	−0.0982 (3)	0.25096 (9)	0.0375 (5)	
H18	0.5414	−0.1462	0.2856	0.045*	
C19	0.58960 (18)	−0.0385 (3)	0.22344 (10)	0.0390 (5)	
H19	0.6579	−0.0447	0.2397	0.047*	
C20	0.56077 (17)	0.0299 (3)	0.17279 (9)	0.0345 (5)	
H20	0.6091	0.0712	0.1546	0.041*	
C21	0.46065 (16)	0.0384 (3)	0.14820 (8)	0.0270 (4)	
C22	0.42919 (16)	0.0983 (3)	0.09300 (9)	0.0277 (4)	
C23	0.33618 (15)	0.0685 (3)	0.06266 (8)	0.0263 (4)	
C24	0.31153 (16)	0.1149 (3)	0.00718 (9)	0.0293 (4)	
C25	0.21394 (16)	0.0753 (3)	−0.02777 (8)	0.0279 (4)	
C26	0.21372 (17)	0.0020 (3)	−0.07754 (9)	0.0322 (5)	
H26	0.2744	−0.0279	−0.0868	0.039*	
C27	0.12542 (19)	−0.0269 (3)	−0.11332 (9)	0.0380 (5)	
H27	0.1252	−0.0804	−0.1466	0.046*	
C28	0.03701 (18)	0.0222 (3)	−0.10051 (10)	0.0368 (5)	
H28	−0.0236	0.0037	−0.1253	0.044*	
C29	0.03689 (17)	0.0983 (3)	−0.05150 (10)	0.0355 (5)	
H29	−0.0237	0.1342	−0.0432	0.043*	
C30	0.12487 (16)	0.1219 (3)	−0.01456 (9)	0.0309 (5)	
H30	0.1245	0.1695	0.0195	0.037*	

### Atomic displacement parameters (Å^2^)

**Table:**

	*U*^11^	*U*^22^	*U*^33^	*U*^12^	*U*^13^	*U*^23^
S1	0.0246 (3)	0.0329 (3)	0.0208 (2)	−0.0011 (2)	0.00740 (19)	0.00065 (19)
S2	0.0320 (3)	0.0295 (3)	0.0255 (3)	−0.0025 (2)	0.0125 (2)	0.0031 (2)
O1	0.0309 (8)	0.0580 (11)	0.0314 (8)	0.0087 (8)	0.0122 (7)	−0.0032 (8)
O2	0.0362 (8)	0.0348 (8)	0.0305 (8)	−0.0102 (7)	0.0072 (7)	0.0043 (7)
O3	0.0214 (7)	0.0516 (10)	0.0303 (8)	0.0001 (7)	0.0057 (6)	−0.0036 (7)
O4	0.0257 (8)	0.0555 (10)	0.0299 (8)	0.0021 (7)	0.0096 (6)	−0.0074 (7)
O5	0.0463 (9)	0.0317 (8)	0.0369 (9)	0.0050 (7)	0.0196 (7)	0.0028 (7)
O6	0.0373 (9)	0.0413 (9)	0.0326 (8)	−0.0091 (7)	0.0133 (7)	0.0082 (7)
O7	0.0328 (8)	0.0504 (10)	0.0343 (9)	−0.0122 (8)	0.0114 (7)	0.0062 (8)
O8	0.0379 (9)	0.0531 (10)	0.0296 (8)	−0.0098 (8)	0.0138 (7)	0.0070 (7)
N1	0.0236 (8)	0.0339 (9)	0.0192 (8)	0.0037 (7)	0.0052 (6)	0.0028 (7)
N2	0.0320 (10)	0.0278 (9)	0.0252 (9)	−0.0069 (8)	0.0101 (7)	0.0008 (7)
C1	0.0305 (10)	0.0254 (10)	0.0206 (9)	−0.0019 (8)	0.0043 (8)	0.0022 (8)
C2	0.0451 (13)	0.0304 (11)	0.0253 (11)	−0.0003 (10)	0.0096 (9)	−0.0007 (9)
C3	0.0595 (16)	0.0334 (12)	0.0246 (11)	−0.0095 (11)	0.0029 (10)	−0.0048 (9)
C4	0.0473 (14)	0.0322 (12)	0.0320 (12)	−0.0120 (10)	−0.0047 (10)	−0.0006 (9)
C5	0.0332 (11)	0.0314 (11)	0.0294 (11)	−0.0074 (9)	0.0011 (9)	0.0053 (9)
C6	0.0290 (10)	0.0226 (10)	0.0237 (10)	−0.0032 (8)	0.0036 (8)	0.0032 (8)
C7	0.0249 (10)	0.0275 (10)	0.0242 (10)	−0.0024 (8)	0.0077 (8)	0.0041 (8)
C8	0.0226 (9)	0.0285 (10)	0.0207 (9)	−0.0001 (8)	0.0064 (7)	0.0025 (8)
C9	0.0239 (10)	0.0294 (10)	0.0247 (10)	−0.0030 (8)	0.0062 (8)	0.0002 (8)
C10	0.0246 (10)	0.0270 (10)	0.0222 (10)	−0.0026 (8)	0.0064 (8)	−0.0020 (8)
C11	0.0304 (11)	0.0291 (10)	0.0257 (10)	−0.0052 (8)	0.0081 (8)	−0.0016 (8)
C12	0.0350 (11)	0.0323 (11)	0.0241 (10)	−0.0024 (9)	0.0025 (9)	0.0039 (8)
C13	0.0295 (11)	0.0357 (12)	0.0313 (12)	−0.0021 (9)	−0.0019 (9)	0.0002 (9)
C14	0.0257 (10)	0.0437 (13)	0.0353 (12)	−0.0075 (10)	0.0067 (9)	−0.0025 (10)
C15	0.0299 (11)	0.0372 (12)	0.0259 (10)	−0.0064 (9)	0.0089 (9)	−0.0007 (9)
C16	0.0327 (11)	0.0238 (10)	0.0262 (10)	−0.0042 (8)	0.0085 (8)	−0.0033 (8)
C17	0.0428 (12)	0.0281 (11)	0.0274 (11)	−0.0034 (9)	0.0098 (9)	0.0017 (9)
C18	0.0494 (15)	0.0318 (12)	0.0282 (11)	−0.0005 (10)	0.0018 (10)	0.0030 (9)
C19	0.0361 (12)	0.0381 (13)	0.0394 (13)	0.0030 (10)	0.0005 (10)	−0.0020 (10)
C20	0.0351 (12)	0.0343 (12)	0.0352 (12)	−0.0036 (9)	0.0103 (10)	−0.0059 (10)
C21	0.0326 (11)	0.0237 (10)	0.0258 (10)	−0.0029 (8)	0.0090 (8)	−0.0026 (8)
C22	0.0316 (11)	0.0262 (10)	0.0282 (11)	−0.0041 (8)	0.0128 (9)	−0.0019 (8)
C23	0.0304 (10)	0.0264 (10)	0.0252 (10)	−0.0027 (8)	0.0128 (8)	0.0000 (8)
C24	0.0344 (11)	0.0288 (11)	0.0270 (11)	−0.0014 (9)	0.0113 (9)	−0.0006 (8)
C25	0.0357 (11)	0.0242 (10)	0.0253 (10)	−0.0021 (8)	0.0101 (9)	0.0043 (8)
C26	0.0359 (12)	0.0363 (12)	0.0261 (11)	0.0039 (9)	0.0107 (9)	0.0013 (9)
C27	0.0462 (14)	0.0391 (13)	0.0272 (11)	0.0012 (11)	0.0050 (10)	−0.0018 (10)
C28	0.0369 (12)	0.0381 (13)	0.0334 (12)	−0.0016 (10)	0.0029 (10)	0.0073 (10)
C29	0.0342 (12)	0.0343 (12)	0.0409 (13)	0.0023 (10)	0.0148 (10)	0.0094 (10)
C30	0.0378 (12)	0.0283 (11)	0.0292 (11)	−0.0003 (9)	0.0130 (9)	0.0017 (9)

### Geometric parameters (Å, °)

**Table:**

S1—O1	1.4283 (16)	C11—C12	1.389 (3)
S1—O2	1.4303 (17)	C11—H11	0.9500
S1—N1	1.6257 (17)	C12—C13	1.382 (3)
S1—C1	1.753 (2)	C12—H12	0.9500
S2—O6	1.4281 (16)	C13—C14	1.391 (3)
S2—O5	1.4296 (17)	C13—H13	0.9500
S2—N2	1.6294 (19)	C14—C15	1.390 (3)
S2—C16	1.757 (2)	C14—H14	0.9500
O3—C7	1.314 (2)	C15—H15	0.9500
O3—H3O	0.97 (3)	C16—C17	1.385 (3)
O4—C9	1.269 (2)	C16—C21	1.404 (3)
O7—C22	1.333 (2)	C17—C18	1.385 (3)
O7—H7O	0.96 (3)	C17—H17	0.9500
O8—C24	1.259 (3)	C18—C19	1.388 (3)
N1—C8	1.429 (2)	C18—H18	0.9500
N1—H1N	0.84 (3)	C19—C20	1.382 (3)
N2—C23	1.436 (3)	C19—H19	0.9500
N2—H2N	0.82 (3)	C20—C21	1.398 (3)
C1—C2	1.389 (3)	C20—H20	0.9500
C1—C6	1.397 (3)	C21—C22	1.467 (3)
C2—C3	1.389 (3)	C22—C23	1.375 (3)
C2—H2	0.9500	C23—C24	1.440 (3)
C3—C4	1.383 (4)	C24—C25	1.486 (3)
C3—H3	0.9500	C25—C30	1.396 (3)
C4—C5	1.388 (3)	C25—C26	1.398 (3)
C4—H4	0.9500	C26—C27	1.382 (3)
C5—C6	1.401 (3)	C26—H26	0.9500
C5—H5	0.9500	C27—C28	1.388 (3)
C6—C7	1.468 (3)	C27—H27	0.9500
C7—C8	1.393 (3)	C28—C29	1.390 (3)
C8—C9	1.433 (3)	C28—H28	0.9500
C9—C10	1.488 (3)	C29—C30	1.388 (3)
C10—C11	1.397 (3)	C29—H29	0.9500
C10—C15	1.400 (3)	C30—H30	0.9500
			
O1—S1—O2	119.62 (10)	C11—C12—H12	120.0
O1—S1—N1	107.65 (10)	C12—C13—C14	120.2 (2)
O2—S1—N1	108.64 (10)	C12—C13—H13	119.9
O1—S1—C1	110.09 (10)	C14—C13—H13	119.9
O2—S1—C1	107.01 (10)	C15—C14—C13	120.4 (2)
N1—S1—C1	102.49 (9)	C15—C14—H14	119.8
O6—S2—O5	119.47 (10)	C13—C14—H14	119.8
O6—S2—N2	107.73 (10)	C14—C15—C10	119.5 (2)
O5—S2—N2	107.98 (10)	C14—C15—H15	120.2
O6—S2—C16	110.37 (10)	C10—C15—H15	120.2
O5—S2—C16	107.55 (10)	C17—C16—C21	122.1 (2)
N2—S2—C16	102.38 (10)	C17—C16—S2	120.70 (17)
C7—O3—H3O	103.1 (15)	C21—C16—S2	117.20 (17)
C22—O7—H7O	103.9 (16)	C16—C17—C18	118.8 (2)
C8—N1—S1	117.40 (14)	C16—C17—H17	120.6
C8—N1—H1N	115.7 (17)	C18—C17—H17	120.6
S1—N1—H1N	114.6 (17)	C17—C18—C19	120.1 (2)
C23—N2—S2	117.47 (15)	C17—C18—H18	120.0
C23—N2—H2N	116.7 (18)	C19—C18—H18	120.0
S2—N2—H2N	114.0 (18)	C20—C19—C18	121.0 (2)
C2—C1—C6	121.9 (2)	C20—C19—H19	119.5
C2—C1—S1	120.28 (17)	C18—C19—H19	119.5
C6—C1—S1	117.65 (15)	C19—C20—C21	120.2 (2)
C1—C2—C3	118.7 (2)	C19—C20—H20	119.9
C1—C2—H2	120.7	C21—C20—H20	119.9
C3—C2—H2	120.7	C20—C21—C16	117.9 (2)
C4—C3—C2	120.4 (2)	C20—C21—C22	120.82 (19)
C4—C3—H3	119.8	C16—C21—C22	121.28 (19)
C2—C3—H3	119.8	O7—C22—C23	121.55 (19)
C3—C4—C5	120.8 (2)	O7—C22—C21	115.35 (19)
C3—C4—H4	119.6	C23—C22—C21	123.07 (18)
C5—C4—H4	119.6	C22—C23—N2	120.07 (18)
C4—C5—C6	119.9 (2)	C22—C23—C24	121.07 (18)
C4—C5—H5	120.1	N2—C23—C24	118.69 (19)
C6—C5—H5	120.1	O8—C24—C23	119.8 (2)
C1—C6—C5	118.30 (19)	O8—C24—C25	117.33 (19)
C1—C6—C7	121.23 (18)	C23—C24—C25	122.79 (18)
C5—C6—C7	120.46 (19)	C30—C25—C26	119.9 (2)
O3—C7—C8	121.67 (19)	C30—C25—C24	122.61 (19)
O3—C7—C6	115.97 (18)	C26—C25—C24	117.28 (19)
C8—C7—C6	122.37 (18)	C27—C26—C25	120.1 (2)
C7—C8—N1	119.84 (18)	C27—C26—H26	119.9
C7—C8—C9	119.69 (18)	C25—C26—H26	119.9
N1—C8—C9	120.35 (17)	C26—C27—C28	120.0 (2)
O4—C9—C8	119.47 (18)	C26—C27—H27	120.0
O4—C9—C10	116.58 (18)	C28—C27—H27	120.0
C8—C9—C10	123.76 (18)	C27—C28—C29	120.2 (2)
C11—C10—C15	119.64 (19)	C27—C28—H28	119.9
C11—C10—C9	116.76 (18)	C29—C28—H28	119.9
C15—C10—C9	123.57 (18)	C30—C29—C28	120.3 (2)
C12—C11—C10	120.3 (2)	C30—C29—H29	119.9
C12—C11—H11	119.8	C28—C29—H29	119.9
C10—C11—H11	119.8	C29—C30—C25	119.5 (2)
C13—C12—C11	119.9 (2)	C29—C30—H30	120.2
C13—C12—H12	120.0	C25—C30—H30	120.2
			
O1—S1—N1—C8	−164.69 (16)	C13—C14—C15—C10	0.9 (4)
O2—S1—N1—C8	64.40 (17)	C11—C10—C15—C14	−0.4 (3)
C1—S1—N1—C8	−48.61 (18)	C9—C10—C15—C14	177.5 (2)
O6—S2—N2—C23	−164.50 (16)	O6—S2—C16—C17	−33.0 (2)
O5—S2—N2—C23	65.20 (18)	O5—S2—C16—C17	98.90 (19)
C16—S2—N2—C23	−48.12 (18)	N2—S2—C16—C17	−147.47 (18)
O1—S1—C1—C2	−36.5 (2)	O6—S2—C16—C21	149.25 (16)
O2—S1—C1—C2	94.97 (19)	O5—S2—C16—C21	−78.84 (18)
N1—S1—C1—C2	−150.81 (18)	N2—S2—C16—C21	34.79 (18)
O1—S1—C1—C6	147.68 (16)	C21—C16—C17—C18	1.4 (3)
O2—S1—C1—C6	−80.85 (18)	S2—C16—C17—C18	−176.28 (17)
N1—S1—C1—C6	33.37 (18)	C16—C17—C18—C19	0.4 (3)
C6—C1—C2—C3	0.8 (3)	C17—C18—C19—C20	−0.8 (4)
S1—C1—C2—C3	−174.87 (17)	C18—C19—C20—C21	−0.6 (4)
C1—C2—C3—C4	−1.3 (4)	C19—C20—C21—C16	2.2 (3)
C2—C3—C4—C5	0.7 (4)	C19—C20—C21—C22	−175.4 (2)
C3—C4—C5—C6	0.5 (3)	C17—C16—C21—C20	−2.7 (3)
C2—C1—C6—C5	0.4 (3)	S2—C16—C21—C20	175.06 (16)
S1—C1—C6—C5	176.13 (16)	C17—C16—C21—C22	174.9 (2)
C2—C1—C6—C7	−180.0 (2)	S2—C16—C21—C22	−7.4 (3)
S1—C1—C6—C7	−4.2 (3)	C20—C21—C22—O7	−15.2 (3)
C4—C5—C6—C1	−1.0 (3)	C16—C21—C22—O7	167.30 (19)
C4—C5—C6—C7	179.4 (2)	C20—C21—C22—C23	163.1 (2)
C1—C6—C7—O3	163.40 (19)	C16—C21—C22—C23	−14.4 (3)
C5—C6—C7—O3	−17.0 (3)	O7—C22—C23—N2	179.2 (2)
C1—C6—C7—C8	−17.3 (3)	C21—C22—C23—N2	1.1 (3)
C5—C6—C7—C8	162.4 (2)	O7—C22—C23—C24	4.1 (3)
O3—C7—C8—N1	−178.91 (18)	C21—C22—C23—C24	−174.0 (2)
C6—C7—C8—N1	1.8 (3)	S2—N2—C23—C22	34.4 (3)
O3—C7—C8—C9	5.1 (3)	S2—N2—C23—C24	−150.31 (17)
C6—C7—C8—C9	−174.25 (19)	C22—C23—C24—O8	−1.1 (3)
S1—N1—C8—C7	35.2 (3)	N2—C23—C24—O8	−176.3 (2)
S1—N1—C8—C9	−148.77 (16)	C22—C23—C24—C25	176.2 (2)
C7—C8—C9—O4	−2.7 (3)	N2—C23—C24—C25	1.0 (3)
N1—C8—C9—O4	−178.73 (19)	O8—C24—C25—C30	−130.8 (2)
C7—C8—C9—C10	172.00 (19)	C23—C24—C25—C30	51.8 (3)
N1—C8—C9—C10	−4.0 (3)	O8—C24—C25—C26	43.7 (3)
O4—C9—C10—C11	33.7 (3)	C23—C24—C25—C26	−133.7 (2)
C8—C9—C10—C11	−141.1 (2)	C30—C25—C26—C27	−1.0 (3)
O4—C9—C10—C15	−144.3 (2)	C24—C25—C26—C27	−175.7 (2)
C8—C9—C10—C15	40.9 (3)	C25—C26—C27—C28	2.1 (4)
C15—C10—C11—C12	−0.3 (3)	C26—C27—C28—C29	−0.9 (4)
C9—C10—C11—C12	−178.4 (2)	C27—C28—C29—C30	−1.5 (3)
C10—C11—C12—C13	0.6 (3)	C28—C29—C30—C25	2.6 (3)
C11—C12—C13—C14	−0.2 (4)	C26—C25—C30—C29	−1.3 (3)
C12—C13—C14—C15	−0.6 (4)	C24—C25—C30—C29	173.0 (2)

### Hydrogen-bond geometry (Å, °)

**Table:**

*D*—H···*A*	*D*—H	H···*A*	*D*···*A*	*D*—H···*A*
N1—H1N···O8	0.84 (3)	2.30 (3)	3.093 (3)	159 (2)
O3—H3O···O4	0.97 (3)	1.55 (3)	2.466 (2)	155 (2)
O7—H7O···O8	0.96 (3)	1.62 (3)	2.510 (2)	153 (3)
C2—H2···O5^i^	0.95	2.57	3.310 (3)	135
C13—H13···O1^ii^	0.95	2.43	3.235 (3)	143
C14—H14···O8^ii^	0.95	2.48	3.396 (3)	162
C15—H15···N1	0.95	2.53	2.990 (3)	110

Symmetry codes: (i) *x*, −*y*+1/2, *z*−1/2; (ii) −*x*+1, −*y*+1, −*z*.
